# Venom IMP-L2 from the Ectoparasitoid *Scleroderma guani* Regulates the IIS/TOR Pathway in *Tenebrio molitor*

**DOI:** 10.3390/insects16080763

**Published:** 2025-07-24

**Authors:** Wenxiu Wang, Zhiquan Zhang, Xuemin Ren, Chaoyan Wu, Jiaying Zhu

**Affiliations:** 1 Key Laboratory of Forest Disaster Warning and Control of Yunnan Province, Southwest Forestry University, Kunming 650224, China; wangwenxiu@swfu.edu.cn (W.W.); jacklinzzq@163.com (Z.Z.); xueminren009@163.com (X.R.); wcy1316033@swfu.edu.cn (C.W.); 2 Key Laboratory for Forest Resources Conservation and Utilization in the Southwest Mountains of China, Ministry of Education, Southwest Forestry University, Kunming 650224, China

**Keywords:** *Scleroderma guani*, venom IMP-L2, IIS/TOR pathways, growth and development

## Abstract

Parasitoid venom significantly influences host physiology and development. Our previous research identified high levels of insulin-binding protein IMP-L2 in the venom of *Scleroderma guani*. IMP-L2 may inhibit the insulin/insulin-like growth factor signaling (IIS) cascade by competitively binding insulin-like peptides (ILPs) with insulin receptor (InR). However, how to regulate IIS transduction is unclear. We speculate that venom-derived IMP-L2 may bind ILPs to inhibit IIS transduction. Consequently, we investigated the regulation of the IIS/TOR pathway by venom-derived IMP-L2. An expression analysis of IIS/TOR pathway genes across various developmental stages of *Tenebrio molitor* demonstrated that this pathway governs the entire developmental process. By examining gene expression before and after parasitism, we determined that *S. guani* predominantly inhibits TOR pathway signaling in *T. molitor* post-parasitism. Bioinformatics and expression analyses revealed that IMP-L2 is critically involved in Hymenoptera insects, exhibiting high expression in the venom apparatus, and is upregulated in response to *S. guani* parasitism factors. Additionally, recombinant IMP-L2 was produced via eukaryotic expression. Finally, the recombinant IMP-L2 was found to inhibit the TOR and IIS/TOR signaling pathways at early (6 h) and late (24 h) stages post-injection. Knockdown of *IMP-L2* in *S. guani* parasitized *T. molitor* pupae, resulting in accelerated death of *T. molitor*. During parasitism, *S. guani* may suppress host growth and development by modulating the IIS/TOR signaling pathway through venom-derived IMP-L2, potentially affecting host lifespan.

## Simple Summary

Most ectoparasitoids delay host development by secreting venom before or during oviposition, thereby ensuring the development of parasitoid eggs. Our previous study found that the venom of the ectoparasitoid *Scleroderma guani* is rich in imaginal morphogenesis factor-Late2 (IMP-L2). In many insects, the IIS/TOR signaling pathway regulates their growth and development. IMP-L2 in Drosophila can inhibit the activation of IIS by binding to insulin-like peptides (ILPs) of the IIS signaling pathway, thereby prolonging the lifespan of Drosophila. However, whether the IIS/TOR signaling pathway plays a role in the growth and development of the host *Tenebrio molitor* and how *S. guani* regulates host IIS/TOR signaling through parasitic behavior and venom IMP-L2 remains unclear. In this study, we found that the IIS/TOR signaling pathway is involved in the growth and development of *T. molitor*, and the parasitic behavior of *S. guani* inhibits the TOR signal transduction of *T. molitor*. IMP-L2 inhibited TOR and IIS/TOR signaling in the early and late stages of injection, respectively. When *IMP-L2* was knocked down, *S. guani* would accelerate the death of *T. molitor* after parasitizing pupae. This study shows that the venom IMP-L2 of *S. guani* may help the egg and larval development of *S. guani* by inhibiting the host IIS/TOR signaling pathway.

## 1. Introduction

Parasitic Hymenoptera represent one of the most diverse and abundant groups of natural enemies within the class Insecta [1]. Currently, it is estimated that over one million species of parasitic wasps play a critical role in the biological control of agricultural pests [2,3]. Endoparasitoids and ectoparasitoids employ distinct strategies, typically utilizing their parasitic factors to inhibit or evade host immune responses while also regulating host growth and development [3]. The parasitic factors associated with these parasitoids include venom, poly DNA viruses (PDVs), virus-like particles (VLPs), and teratocytes [4]. Among these, venom is the most prevalent and extensively studied parasitic factor, containing a complex mixture of proteins and non-protein active compounds [1,5], many of which remain unidentified. In most ectoparasites, the primary function of venom is to induce paralysis or lethargy and developmental arrest in the host, particularly during the larval or nymphal stages [6]. Most parasitoids administer venom into the host prior to or during oviposition to manipulate host physiology, thereby facilitating the development of parasitoid eggs [7].

In insects, the insulin/insulin-like growth factor and target of rapamycin signaling pathways (IIS/TOR) coordinate metabolic processes by integrating nutrient and hormonal signals, thereby influencing developmental outcomes [8,9,10]. Each signaling molecule within the IIS/TOR pathway plays a vital regulatory role [11,12]. Insulin-like peptides (ILPs) are predominantly produced in the medial neurosecretory cells of the brain, as well as in the ovary, fat bodies, glial cells, and midgut [13,14,15,16,17,18]. Ablation of ILP-secreting neuroendocrine cells can lead to significant reductions in insect body size and manifestations resembling diabetes [19]. ILP secretion activates the IIS cascade by binding to and activating insulin receptor 1 (InR1), which in turn activates the insulin receptor substrate protein (CHICO). This InR1-mediated signaling transduces to phosphatidylinositol 3-kinase (PI3K), the phosphoinositide-dependent protein kinase 1 (PDK) and protein kinase B (AKT), inhibiting the forkhead box class O transcription factor (FOXO), a crucial downstream target of IIS, thereby regulating energy metabolism, reproduction, and longevity in insects [20,21,22,23,24]. The phosphatase and tensin homolog (PTEN) downregulates PI3K/PDK signaling, ultimately inhibiting the IIS pathway [25,26]. Additionally, the second insulin receptor, InR2, can inhibit InR1, and the co-expression of InR1 and InR2 allows FOXO to regulate transcription within the nucleus [23]. Overexpression of FOXO may impede early larval growth and development, resulting in smaller larvae [27,28], while the absence of FOXO is associated with delayed adult development and reduced body size [29]. Therefore, FOXO is essential for the regulation of insect growth and development. Moreover, AKT can inhibit the negative regulators of the TOR pathway, tuberous sclerosis complex 1 and 2 (TSC1/2), thereby activating downstream ras homolog enhanced in brain (RHEB)/TOR signaling [30,31]. Elevated intracellular amino acid concentrations also enhance TOR activity via RHEB [30,32]. Active TOR promotes cell growth by inhibiting the translational repressor 4e-binding protein (4EBP) and stimulating ribosomal protein S6 kinase (S6K), thereby facilitating translation and ribosome biogenesis [22]. Activation of 4EBP and inhibition of S6K1 can extend insect lifespan [33]. Furthermore, under high amino acid conditions, TOR signaling can activate GATA activator subtypes, leading to the activation of ILP1, ILP2, ILP3, ILP5, and ILP8 in *Aedes aegypti*, thus stimulating the IIS pathway [34]. Simultaneously, TOR also regulates AKT expression, while S6K modulates CHICO expression and PDK phosphorylation, thereby influencing IIS signal transduction [35,36,37].

An insulin-like peptide (DILPs) binding protein, imaginal morphogenesis factor-Late2 (IMP-L2), has been identified in *Drosophila* [38]. IMP-L2 belongs to the immunoglobulin superfamily and has been shown to bind human insulin-like growth factors I and II (IGF-I and IGF-II) as well as insulin with high affinity [39]. Initially considered an ortholog of insulin-like growth factor binding proteins (IGFBPs) [40,41], recent findings indicate that the structure of IMP-L2 and its hormone binding characteristics do not significantly correlate with human IGFBPs [42], leaving this classification controversial. Overexpression of IMP-L2 in *Drosophila* leads to a twofold increase in mRNA levels of *DILP2*, *DILP3*, and *DILP5* [43]. Concurrently, secreted IMP-L2 binds to native DILP-2 and DILP-5, acting as a negative regulator of IIS activity and extending *Drosophila* lifespan [43,44,45].

The expression of IMP-L2 is influenced by nutritional status; under conditions of malnutrition, its expression in the fat body increases, thereby inhibiting overall IIS activity and enabling the organism to withstand starvation [45,46]. Fruit flies deficient in IMP-L2 not only exhibit larger body sizes but also resist size reduction under nutritional scarcity [46]. Notably, the regulation of nutrient-dependent IMP-L2 expression is mediated through ecdysone signaling rather than the TOR nutrient sensor in the fat body [46]. Elevated levels of ecdysone stimulate the release of IMP-L2 from the fat body [46]. Additionally, IMP-L2 expression in the intestine and specific neurons contributes to the regulation of the IIS. Enhanced expression of IMP-L2 in the intestine can inhibit IIS and promote lifespan extension, whereas its expression in certain neurons is crucial for maintaining normal IIS activity in the brain and associated glands [43,47,48]. Furthermore, overexpression of IMP-L2 can induce a 100% diapause response via the neuroendocrine cell driver c929 [49].

In summary, parasitic wasps secrete venom that influences host physiology, thereby affecting host growth and development. The IIS/TOR signaling pathway is known to regulate these processes; however, there is currently no research addressing the role of the IIS/TOR signaling pathway in the growth and development of *T. molitor*. Additionally, IMP-L2, derived from non-venom sources across various tissues, negatively regulates the IIS/TOR signaling pathway by binding to insulin-like peptides (ILPs). However, the regulatory mechanism is not clear. Previous studies have identified a high concentration of IMP-L2 in the venom of *S. guani* [50], but it has only been reported in the venoms of *Nasonia vitripennis* and *Eumenes pomiformis* [51,52]. Notably, the impact of venom-derived IMP-L2 on the host IIS/TOR signaling pathway remains unexplored.

This study aims to investigate the expression characteristics of the IIS/TOR signaling pathway throughout the developmental stages of *T. molitor* and elucidate how this pathway regulates the growth and development of the species. Additionally, the research will examine how parasitism influences the IIS/TOR signaling pathway in *T. molitor*, determining whether parasitic wasps alter host growth and development through modulation of this signaling route. Furthermore, we will analyze the sequence, tissue expression, and parasitic responses of IMP-L2 to clarify its evolutionary biology and expression patterns. Finally, the study will investigate the effects of venom-derived IMP-L2 on the IIS/TOR signaling pathway in *T. molitor*, providing a theoretical framework for understanding the influence of venom IMP-L2 on the growth and development of this beetle species.

## 2. Materials and Methods

### 2.1. Insects

Following the methodology outlined by Zhu [53], *T. molitor* were maintained on wheat bran under natural light conditions and at room temperature, with hydration provided through Chinese cabbage. Adult *S. guani* were nourished with a 20% honey–water solution, and the subsequent generation of *S. guani* was propagated by allowing adults to parasitize fresh pupae of *T. molitor*.

### 2.2. The Expression of IIS/TOR Pathway Related Genes in Different Developmental Stages of T. molitor

A total of 100 early-stage (2-day-old) eggs, 100 mid-stage (4-day-old) eggs, and 100 late-stage (6-day-old) eggs, along with 50 newly hatched larvae (one instar larvae), old larvae (7–8 instar larvae), last instar larvae (14 instar larvae), prepupae, early pupae (white pupae), mid-stage pupae (yellow pupae), late pupae (black pupae), and seven adults at various ages (1, 4, 8, 12, 16, 20, and 24 days post-eclosion) were collected and subjected to disinfection. RNA extraction and cDNA synthesis for each sample were performed according to Wang’s protocol [54]. Primers targeting cDNA sequences of genes related to the IIS/TOR pathway were designed (Table S1). Ribosomal protein L32 (*rpl*) was utilized as an internal reference, and RT-qPCR was conducted using SYBR^®^ Premix Ex Taq II (Takara Biomedical Technology Co., Ltd., Beijing, China) in a 20 μL reaction volume. The RT-qPCR conditions included an initial denaturation at 95 °C for 1 min, followed by 45 cycles of denaturation at 95 °C for 15 s and annealing at 60 °C for 60 s. The experiment was replicated three times.

### 2.3. Regulation of Parasitism on Genes Related to IIS/TOR Signaling Pathway in T. molitor

Healthy *T. molitor* pupae, weighing between 0.9 and 1.4 mg, were sterilized using absorbent cotton dipped in 75% alcohol under sterile conditions. *S. guani* and *T. molitor* pupae were placed in finger-shaped glass tubes, which were sealed with cotton balls to facilitate the observation of *S. guani* parasitism on *T. molitor* pupae. Timing commenced when the tail of *S. guani* penetrated the pupae, after which the parasitized pupae were individually transferred to a constant temperature incubator. Pupae parasitized by one female and one male were collected at 6 h and 24 h, respectively, and RNA and cDNA were extracted from each sample according to Wang’s protocol [54]. Unparasitized pupae from the same time period served as controls. The reference gene for RT-qPCR, along with the primers for the IIS/TOR pathway genes, reaction system, and procedures were as described in Section 2.2. The entire experiment was replicated three times.

### 2.4. Cloning and Sequence Analysis of IMP-L2 from S. guani

Following the aforementioned method, RNA and cDNA from *S. guani* were extracted, and the full-length coding sequence (cds) of *IMP-L2* was amplified using RT-PCR primers listed in Table S1. The amplified product was then ligated into the commercial vector pEASY^®^-T1 Simple Cloning Kit (TransGen Biotech Co., Ltd., Beijing, China) and transformed into competent *E. coli* DH5α cells. Positive transformants were sent to Platinum Biotechnology Co., Ltd. (Shanghai, China) for sequencing. The resulting sequences were processed using DNAstar Lasergene 11.0 software (DNASTAR Inc., Madison, WI, USA), and the assembled nucleic acid sequences were analyzed for homology through a BlastX search in NCBI (https://blast.ncbi.nlm.nih.gov, accessed on 16 September 2024). Subsequent analyses, including translation, signal peptide prediction, multiple sequence alignment, phylogenetic tree construction, and physical and chemical properties assessment, were performed using DNAMAN (Lynnon Biosoft Inc., San Ramon, CA, USA), SignalP 6.0 (https://services.healthtech.dtu.dk/services/SignalP-6.0/, accessed on 16 September 2024), CLUSTAL (https://www.ebi.ac.uk/jdispatcher/, accessed on 16 September 2024), Mega 7.0 (1000 neighbor-joining method) (Temple University, Philadelphia, PA, USA), ProtParam (https://web.expasy.org/protparam/, accessed on 16 September 2024), and Scansite 4.0 (https://scansite4.mit.edu/#scanProtein, accessed on 16 September 2024) for domain prediction.

### 2.5. Expression Analysis of IMP-L2 in S. guani Venom Organs, Developmental Stages, and Parasitic Process

Various tissues (venom apparatus and abdominal residues) were collected from *S. guani* across four developmental stages (15 periods: one egg period; four larval periods; three pupae periods; seven adult periods), including both parasitized and unparasitized samples, under an anatomical microscope. Female wasps that had mated for the same duration were placed in a finger-shaped tube containing fresh pupae of *T. molitor*. Wasps exhibiting piercing behavior and that paralyzed the host were classified as parasitized; those that did not exhibit this behavior were deemed unparasitized. RNA and cDNA extraction for each sample followed Wang’s method [54]. For the different developmental stages of *S. guani*, *18S rRNA* served as the reference gene for the *IMP-L2* gene, while *5.8S rRNA* was used as the reference in abdominal residues and venom organs in both parasitic and non-parasitic conditions. The primers for *IMP-L2*, *18S rRNA*, and *5.8S rRNA* used in RT-qPCR are detailed in Table S1, with the reaction system and procedures referenced in Section 2.2. A total of 80 venom apparatus and abdominal residues were analyzed, along with 50 wasps from different developmental stages, respectively, and 40 female wasps from each of the parasitized and unparasitized wasps. The entire experiment was conducted in triplicate.

### 2.6. Construction of the Expression Vector pFast-bac1-IMP-L2 and Recombinant Baculovirus Expression Vector (Bacmid)

Based on the cloned *IMP-L2* gene sequence of *S. guani* venom, the target gene was synthesized by PAS (PCR-based Accurate Synthesis) method. The target gene was digested by BamH I and Hind III and ligated to the pFast-bac1 vector with His tag to obtain the plasmid pFast-bac1-*IMP-L2*. The upstream/downstream primer of its digestion in Table S1.

The obtained recombinant plasmid was transferred into *E. coli* TOP10 competent cells, and the positive clones were selected and sent to Nanjing Zhong ding Biotechnology Co., Ltd. (Nanjing, China) for sequencing verification.

The sequencing-confirmed 200 ng pFast-bac1-*IMP-L2* vector and pFast-bac1 empty vector were, respectively, added to DH10Bac *E. coli* competent cells, gently mixed, and placed on ice for 30 min. After a 42 °C heat shock for 90 s, they were quickly placed on the ice for 5 min. After that, a 900 μL SOC medium was added to the EP tube of activated competent cells and shaken in a shaker for 4 h at 37 °C at 225 rpm. The LB plate (containing gentamicin 7 ug/mL, kanamycin 50 ug/mL, tetracycline 10 ug/mL, and then an added 10 μL 24 mg/mL IPTG and 40 μL 20 mg/mL x-gal) was smeared with 100 μL of the bacterial solution and cultured at 37 °C for 48 h. Positive clones were chosen and stored at −80 °C.

### 2.7. Expression and Purification of the IMP-L2 Protein

A total of 2 mL of Sf9 cells (*Spodoptera frugiperda*) at a concentration of 9 × 10^5^ cells per mL were transferred to each well of a six-well plate and cultured at 27 °C for 1 h. Subsequently, 1 µg of bacmid (pFast-bac1-*IMP-L2* or pFast-bac1, approximately 5 µL) was diluted in 100 µL of Grace’s medium, devoid of both antibiotics and fetal bovine serum (FBS). Additionally, 6 µL of Cellfectin Reagent (Thermo Fisher Scientific Inc., Wilmington, MA, USA) was diluted with 100 µL of incomplete Grace’s medium, also devoid of antibiotics and FBS. The two solutions were combined, gently mixed, and incubated at room temperature for 15 to 45 min.

Next, 800 µL of the incomplete Grace’s culture medium was added to the mixture containing the bacmid and Cellfectin Reagent, which was then gently mixed and distributed into each well. The cells in the six-well plate were incubated at the same temperature for 5 h, after which the mixture was removed. Two mL of complete medium was added to each well, and the incubation continued for 72 h or until signs of viral infection were observed.

Upon observing signs of infection, the culture supernatant was transferred to a 15 mL centrifuge tube and centrifuged at 1000 rpm for 5 min at 4 °C to eliminate cell debris, yielding the cell supernatant containing the P1 virus. A suitable volume of this supernatant was inoculated into a 50 mL shake flask, and the supernatant was collected 72 h post-infection.

The cell supernatant was diluted 100-fold and dialyzed overnight against PBS buffer (pH 7.4) (Sangon Biotech Co., Ltd., Shanghai, China). The dialyzed supernatant was subsequently incubated with a balanced Ni column in a rotary incubator at 4 °C for 3–4 h. Following this, the incubated sample was gradually applied to a low-pressure chromatography system equipped with a Ni-IDA-Sepharose CL-6B affinity chromatography column (Sangon Biotech Co., Ltd., Shanghai, China), using a binding/wash buffer (pH 7.9–8.1) (Sangon Biotech Co., Ltd., Shanghai, China) at a flow rate of 0.5 mL/min until the OD_280_ value of the effluent returned to baseline.

The protein solution was then eluted with Ni-IDA elution buffer (pH 7.9–8.1) (Sangon Biotech Co., Ltd., Shanghai, China), collected at a flow rate of 1 mL/min, and transferred to a dialysis bag, where it was dialyzed overnight at 4 °C with PBS buffer (pH 7.4) (Sangon Biotech Co., Ltd., Shanghai, China) to obtain purified IMP-L2 and His-tag proteins.

### 2.8. SDS-PAGE and Western Blot Analysis

The purified protein solution was analyzed using 10% SDS-PAGE, and the results were visualized following staining with Coomassie Brilliant Blue and subsequent decolorization. For Western blot analysis, 10 µL of the dialysis-purified protein sample was loaded into the wells of the polyacrylamide gel. The concentrated gel was run at 90 V initially, followed by a separation gel run at 200 V. After electrophoresis, the gel was removed, and protein transfer to the membrane was performed at 100 V for 1.5 h. The membrane was washed with PBS four times for 5 min each.

Next, the membrane was incubated in 5% skim milk powder solution (blocking solution) at 37 °C for 1 h to block nonspecific binding. The 30 mL blocking solution containing His-Tag (2A8) antibody (1:5000; Abmart Co., Ltd., Shanghai, China) against the His-tag protein was then diluted in the blocking solution and applied to the membrane for 1 h at 37 °C. After this incubation, the membrane was washed four times, with each wash lasting 5 min. The goat anti-mouse IgG (1:10,000; Abmart Co., Ltd., Shanghai, China) was similarly diluted in the blocking solution and incubated with the membrane at 37 °C for 1 h.

Finally, after rinsing the membrane, enhanced chemiluminescence (ECL) imaging was conducted, and the resulting gel image served as the experimental outcome.

### 2.9. The Regulation of Recombinant IMP-L2 Protein on the Genes Related to the IIS/TOR Signaling Pathway of T. molitor

The expressed recombinant IMP-L2 protein was diluted to a concentration of 0.125 µg/µL (the content of IMP-L2 in a venom reservoir is 0.125 μg) in PBS (pH 7.4). Disinfection of *T. molitor* pupae was performed prior to experimentation. Following the duration of parasitic treatment, 2 µL of recombinant IMP-L2 was extracted using a 10 µL micro-injector (Gaoge Industry and Trade Co., Ltd., Shanghai, China) and injected into the penultimate abdominal segment of the pupae. A small amount of petroleum jelly was applied to the injection site, and the treated pupae were subsequently placed in a temperature-controlled incubator.

Following the protocol outlined in Section 2.3, the injected pupae were paired with one male and one female at 6 h and 24 h post-injection, respectively. The reference genes for RT-qPCR, along with primers for IIS/TOR pathway genes, reaction system, and procedures, were based on the methodology described in Section 2.2. His-tag protein was injected as a control, with a total of 20 heads subjected to injection for each treatment group. The entire experiment was conducted in triplicate.

### 2.10. Synthesis of dsRNA and RNA Interference

Specific primers (Table S1) containing the T7 promoter were designed based on the cDNA sequences of IMP-L2 (Genbank: PP108620) and GFP (from the pEGFP-1 plasmid preserved in the laboratory). A 2× Phanta Max Master Mix (Dye Plus) (Vazyme Biotech Co., Ltd., Nanjing, China) was used for PCR amplification. The amplified products were purified by SteadyPure Agarose Gel DNA Purification Kit (Accurate Biotechnology (Hunan) Co., Ltd., Changsha, China). The purified products were used to synthesize dsIMP-L2 (double-stranded IMP-L2 RNA) and dsGFP (double-stranded GFP RNA) by T7 RiboMAX TM Express RNAi System (Promega Biotech Co., Ltd., Beijing, China). The concentration of the synthesized double-stranded RNA was detected by NanoDrop One (Thermo Fisher Scientific Inc., Wilmington, MA, USA). The dsRNA was diluted to 5000 ng/uL with RNase Free H_2_O and placed at −80 °C.

The newly emerged female *S. guani* was used for RNA interference experiments. First, 200 nL (1 ug) dsRNA was injected at a rate of 20 nL/s with Nanoject II Programmable Nanoliter Injector (Drummond Scientific Company, Broomall, PA, USA). After injection, *S. guani* was reared in an incubator at 28 ± 2 °C and relative humidity of 60–70% (fed with honey water). After 24 h of injection, Trizol was used to extract RNA from the experimental group and the control group. The extracted total RNA was used to synthesize cDNA with HiScript III RT SuperMix for qPCR (+gDNA wiper) (Vazyme Biotech Co., Ltd., Nanjing, China). RT-qPCR primers were designed, and 5.8S rRNA gene was used as the internal reference gene (Table S1). The expression level of *IMP-L2* was verified by RT-qPCR. The dsIMP-L2 was used as the experimental group and dsGFP was used as the control group. Each group consisted of 3–5 *S. guani*, and the experiment included three biological replicates.

### 2.11. Records of Survival Pupae of T. molitor

The dsGFP and dsIMP-L2 were injected into 30 newly emerged *S. guani* females, respectively, and a treated *S. guani* parasitized an early *T. molitor* pupae. After observation of the parasitic behavior, the *S. guani* was removed, and the parasitized *T. molitor* pupae were placed in an incubator at 28 ± 2 °C and relative humidity of 60–70%. The time from parasitism to death of *T. molitor* pupae was recorded (the successfully parasitized pupae could not eclosion).

### 2.12. Statistical Analyses

Real-time fluorescence quantitative data were analyzed using the 2^(−∆∆CT)^ method [55]. Data analysis was performed with IBM SPSS Statistics 20.0 (SPSS Inc., Chicago, IL, USA), employing one-way ANOVA and the Tukey’s multiple-comparisons test to assess significant differences in the relative expression of IIS/TOR signaling pathway genes or *IMP-L2* gene across various developmental stages of *T. molitor* or *S. guani*. Differences in the relative expression levels of *IMP-L2* among different tissues of *S. guani*, as well as between parasitized versus and unparasitized conditions, were evaluated using Student’s *t*-test. Additionally, significant differences in the relative expression of *T. molitor* IIS/TOR signaling pathway genes between *S. guani* under parasitized and unparasitized conditions at 6 h and 24 h were also assessed using Student’s *t*-test. The comparative expression levels of *T. molitor* IIS/TOR signaling pathway genes between recombinant IMP-L2 and His-tag injections at 6 h and 24 h were similarly analyzed. The interference efficiency of dsGFP and dsIMP-L2 after interference was also compared using the same analysis. Statistical significance was determined at *p* < 0.05. Graphical representations were created using GraphPad Prism 9.5 (GraphPad Software Inc., San Diego, CA, USA) and Photoshop CS6 (Adobe Systems Inc., San Jose, CA, USA).

## 3. Results

### 3.1. The Expression Pattern Analysis of IIS/TOR Signaling Pathway Related Genes in Different Developmental Stages of T. molitor

**Figure 1:**
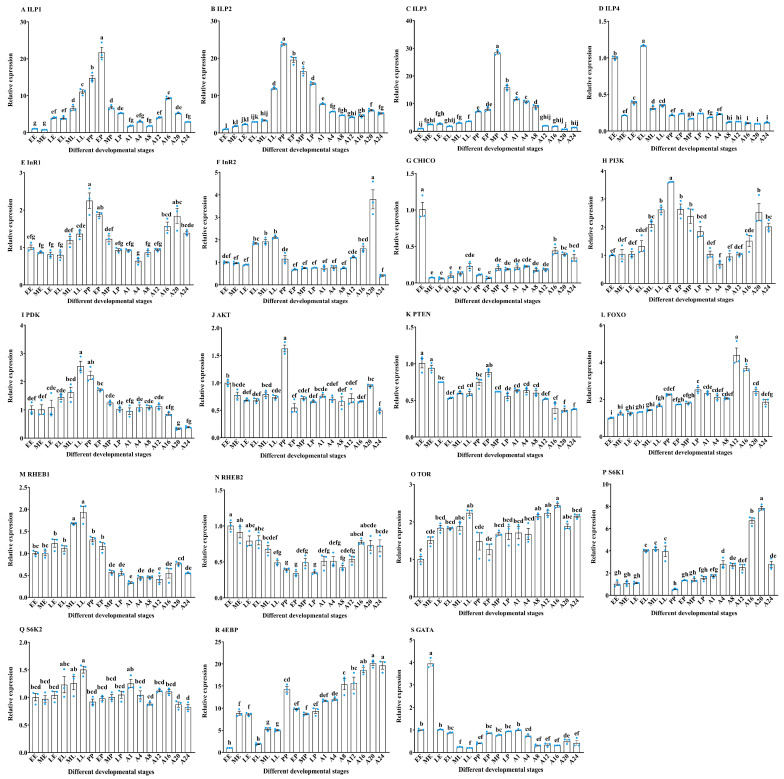
The expression of genes related to IIS/TOR signaling pathway in different developmental stages of *T. molitor*. (**A**–**D**) Expression of insulin-like peptide (*ILP*)-1/2/3/4; (**E**,**F**) expression of insulin receptor (*InR*)-1/2; (**G**) expression of insulin receptor substrate (*CHICO*); (**H**) expression of phosphatidylinositol 3-kinase (*PI3K*); (**I**) expression of phosphoinositide-dependent protein kinase (*PDK*); (**J**) expression of protein kinase B (*AKT*); (**K**) expression of phosphatase and tensin homolog (*PTEN*); (**L**) expression of transcription factor forkhead box class O (*FOXO*); (**M**,**N**) expression of ras homolog enhanced in brain (*RHEB*)-1/2; (**O**) expression of target of rapamycin (*TOR*); (**P**,**Q**) expression of stimulating ribosomal protein S6 kinase (*S6K*)-1/2; (**R**) expression of translational repressor 4e-binding protein (*4EBP*); (**S**) expression of GATA transcription factor (*GATA*) in different developmental stages. EE, early egg; ME, middle egg; LE, later egg; EL, early larvae; ML, middle larvae; LL, later larvae; PP, prepupae; EP, early pupae; MP, middle pupae; LP, later pupae; A1, adult after 1 day of emergence; A4, adult after 4 days of emergence; A8, adult after 8 days of emergence; A12, adult after 12 days of emergence; A16, adult after 16 days of emergence; A20, adult after 20 days of emergence; A24, adult after 24 days of emergence. Means ± standard error (means ± SE) corresponding to different letters within a same gene was significantly different (*p* < 0.05, Tukey’s multiple comparison test), while solid circles indicate values for individual biological replicates.

The IIS/TOR signaling pathway plays a critical role in regulating insect growth and development [10]. To investigate its involvement throughout the developmental stages of *T. molitor*, we assessed the expression of genes associated with this pathway across various developmental phases. RT-qPCR results indicated that *ILP* genes were expressed during different developmental stages of *T. molitor*, including three egg stages (EE, ME, LE), three larval stages (EL, ML, LL), one prepupal stage (PP), three pupal stages (EP, MP, LP), and seven adult stages (A1, A4, A8, A12, A16, A20, A24). The expression of *ILP1* increased progressively from the EE to EP stage, peaked in the EP stage, and subsequently declined (F_16,34_ = 152.66, *p* < 0.001) (Figure 1A). In contrast, *ILP2* expression was markedly elevated during the pupal stage compared to the other stages, with the highest levels detected in the PP stage (F_16,34_ = 574.45, *p* < 0.001) (Figure 1B). Furthermore, *ILP2* expression exhibited an upward trend from EE to LL, declined from PP to LP, and remained stable from A1 to A24 (Figure 1B). Similarly, *ILP3* expression was significantly higher during the pupal and early adult stages (A1–A8), peaking in the MP stage (F_16,34_ = 601.33, *p* < 0.001) (Figure 1C), while expression remained stable in the egg, larval, and late adult stages (A12–A24) (Figure 1C).

In contrast to the elevated expression of *ILP1–3* during the pupal stage, *ILP4* expression was significantly higher in the EL and EE stages compared to all other developmental stages (F_16,34_ = 1338.05, *p* < 0.001) (Figure 1D). The *InR1* gene exhibited a gradual increase from the EE to PP stage, followed by a decline to A4, with a second peak at A20 (F_16,34_ = 23.88, *p* < 0.001). Meanwhile, *InR2* showed relatively high expression during the larval stage, peaking at A20 (F_16,34_ = 47.32, *p* < 0.001) (Figure 1E,F). The *CHICO* gene had the highest expression at EE, gradually increasing from ME to LL, and remaining stable from MP to A12 (F_16,34_ = 48.84, *p* < 0.001) (Figure 1G). Reflecting the pattern of *InR1*, *PI3K* expression increased from EE to PP, peaked in the PP stage, decreased to A4, and then increased again from A4 to A20 (F_16,34_ = 28.43, *p* < 0.001) (Figure 1H). The *PDK* gene exhibited a gradual increase from EE to LL, with no significant difference between LL and PP stages, followed by a decline (F_16,34_ = 22.46, *p* < 0.001) (Figure 1I). The *AKT* gene expression peaked in the PP stage (F_16,34_ = 29.58, *p* < 0.001) (Figure 1J). The *PTEN* gene was highly expressed during the egg stage and increased in the PP stage, though it remained lower than in the egg stage (F_16,34_ = 30.96, *p* < 0.001) (Figure 1K). The *FOXO* gene demonstrated continuous upregulation after the EE stage, with heightened expression in the A12 and A16 stages (F_16,34_ = 56.14, *p* < 0.001) (Figure 1L). The *RHEB1* gene was highly expressed in the LL stage (F_16,34_ = 57.38, *p* < 0.001), while *RHEB2* peaked in the EE stage (F_16,34_ = 14.58, *p* < 0.001), and *TOR* expression was relatively high in the LL, A12, and A16 stages (F_16,34_ = 11.56, *p* < 0.001) (Figure 1M–O). The S6K gene expression in *T. molitor* developmental stages was responsive to TOR signal transduction. Specifically, *S6K1* was more highly expressed during the larval and adult stages, peaking at A20 (F_16,34_ = 102.68, *p* < 0.001) (Figure 1P), while *S6K2* was highly expressed in the LL stage (F_16,34_ = 6.31, *p* < 0.001) (Figure 1Q). The *4EBP* gene exhibited a gradual increase from the LP to A24 stage, with high expression in the PP stage (F_16,34_ = 97.19, *p* < 0.001), whereas *GATA* expression peaked in the ME stage (F_16,34_ = 305.45, *p* < 0.001) (Figure 1R,S).

### 3.2. Expression Pattern Analysis of Genes Related to the IIS/TOR Signaling Pathway of T. molitor in Response to S. guani Parasitism

The above studies have shown that the IIS/TOR signaling pathway plays a role in various stages of *T. molitor* development. Given that parasitic wasps can influence the growth and development of their hosts, this study aimed to assess the impact of parasitism on the IIS/TOR signaling pathway in *T. molitor*.

**Figure 2:**
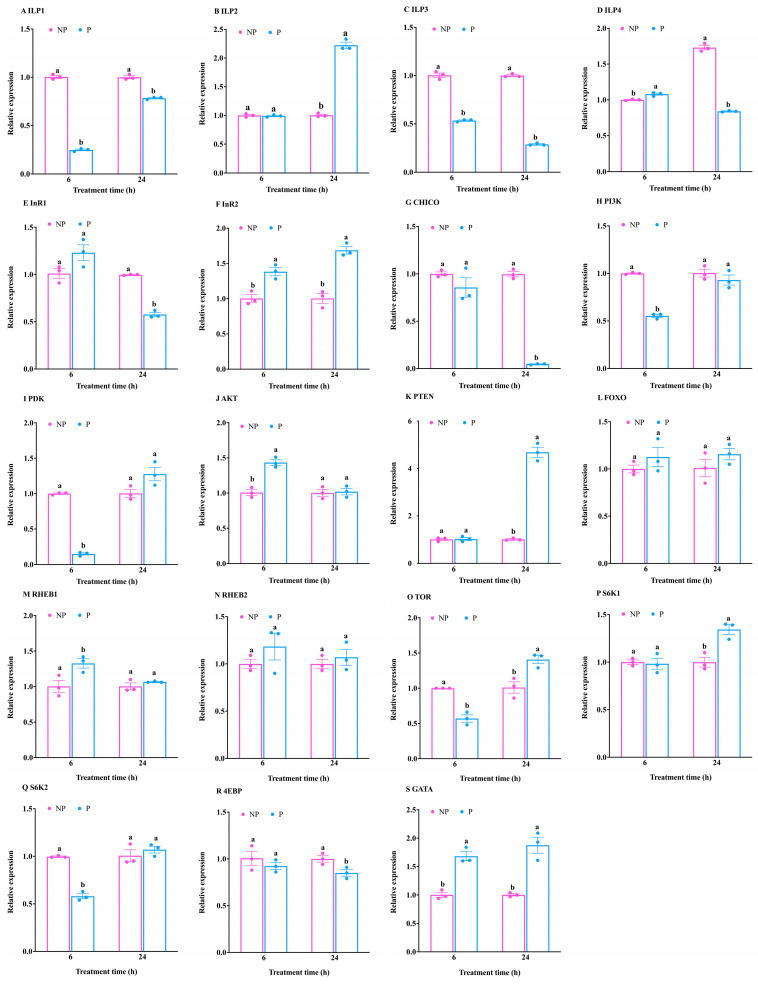
Effect of parasitization by *S. guani* on the expression of IIS/TOR signaling related genes of *T. molitor*. (**A**–**D**) Expression of insulin-like peptide (*ILP*)-1/2/3/4; (**E**,**F**) expression of insulin receptor (*InR*)-1/2; (**G**) expression of insulin receptor substrate protein (*CHICO*); (**H**) expression of phosphatidylinositol 3-kinase (*PI3K*); (**I**) expression of phosphoinositide-dependent protein kinase (*PDK*); (**J**) expression of protein kinase B (*AKT*); (**K**) expression of phosphatase and tensin homolog (*PTEN*); (**L**) expression of transcription factor forkhead box class O (*FOXO*); (**M**,**N**) expression of ras homolog enhanced in brain (*RHEB*)-1/2; (**O**) expression of target of rapamycin (*TOR*); (**P**,**Q**) expression of stimulating ribosomal protein S6 kinase (*S6K*)-1/2; (**R**) expression of translational repressor 4e-binding protein (*4EBP*); (**S**) expression of GATA transcription factor (*GATA*) at different time under parasitized and non-parasitized conditions. P, parasitization; NP, non-parasitization. Means ± standard error (means ± SE) corresponding to different letters within a same gene at same time was significantly different (*p* < 0.05, Student’s *t* test) while solid circles indicate values for individual biological replicates.

At 6 h post-parasitism, significant reductions in the expression levels of the *ILP1* and *ILP3* genes were observed in *T. molitor* compared to the control group (*ILP1*: t = −44.52, df = 4, *p* < 0.001; *ILP3*: t = −19.37, df = 4, *p* < 0.001) (Figure 2A,C). Conversely, *ILP4* expression was significantly elevated in the parasitized group (t = 4.90, df = 4, *p* < 0.01) (Figure 2D). Additionally, the expression of the *InR2* gene was upregulated following parasitism (t = −4.79, df = 4, *p* < 0.001) (Figure 2F).

Moreover, parasitism was found to suppress the expression of the *PI3K* and *PDK* genes (*PI3K*: t = 25.94, df = 4, *p* < 0.001; *PDK*: t = 52.23, df = 4, *p* < 0.001), while the *AKT* gene exhibited upregulation (t = −7.56, df = 4, *p* < 0.01) (Figure 2H–J). The *RHEB1* gene within the TOR signaling pathway was transcriptionally induced in response to the elevated *AKT* expression (t = −2.98, df = 4, *p* < 0.05) (Figure 2M). However, the expression of the *TOR* gene and *S6K2* gene was significantly suppressed (*TOR*: t = 8.39, df = 4, *p* < 0.01; *S6K2*: t = 15.65, df = 4, *p* < 0.001) (Figure 2O,Q), while *GATA* expression was markedly increased (t = −7.40, df = 4, *p* < 0.01) (Figure 2S).

Importantly, there was no observed effect on the transcription levels of *ILP2*, *InR1*, *CHICO, PTEN, FOXO, RHEB2, S6K1*, and *4EBP* genes (*ILP2*: t = −0.48, df = 4, *p* > 0.05; *InR1*: t = −2.23, df = 4, *p* > 0.05; *CHICO*: t = 1.35, df = 2.19, *p* > 0.05; *PTEN*: t = −0.14, df = 4, *p* > 0.05; *FOXO*: t = −1.16, df = 4, *p* > 0.05; *RHEB2*: t = −1.22, df = 4, *p* > 0.05; *S6K1*: t = 0.28, df = 4, *p* > 0.05; *4EBP*: t = 0.96, df = 4, *p* > 0.05) (Figure 2B,E,G,K,L,N,P,R). These results suggest that parasitism for 6 h minimally affects the IIS signaling pathway, while exerting a significant inhibitory influence on the TOR pathway.

At 24 h after parasitism, the expression levels of *ILP1*, *ILP3,* and *ILP4* genes in *T. molitor* were significantly lower than those in the control group (*ILP1*: t = −13.00, df = 4, *p* < 0.001; *ILP3*: t = −59.35, df = 4, *p* < 0.001; *ILP4*: t = −27.25, df = 4, *p* < 0.001); the expression level of *ILP2* gene was significantly higher than that of the control group (t = 21.60, df = 4, *p* < 0.001) (Figure 2A–D). In addition, the expression of *InR1* gene was inhibited (t = 17.69, df = 4, *p* < 0.001) and the expression of *InR2* gene was induced (t = −8.03, df = 4, *p* < 0.01) after 24 h of parasitism (Figure 2E,F).

At the same time, the expression of *CHICO* was inhibited (t = 32.98, df = 4, *p* < 0.001) (Figure 2G), and the expression of *PTEN* gene was upregulated (t = −16.95, df = 4, *p* < 0.001) (Figure 2K), which affected the InR1/CHICO signal transduction, but the expression of *FOXO*, *PI3K*, *PDK,* and *AKT* genes was not affected (*FOXO*: t = −1.33, df = 4, *p* > 0.05; *PI3K*: t = 1.02, df = 4, *p* > 0.05; *PDK*: t = −2.45, df = 4, *p* > 0.05; *AKT*: t = −0.22, df = 4, *p* > 0.05) (Figure 2H–J,L). The expression of *TOR* gene was upregulated (t = −3.97, df = 4, *p* < 0.05) (Figure 2O), and the downstream signal transduction was activated, so that the expression of *S6K1* and *GATA* genes was significantly upregulated (*S6K1*: t = −4.68, df = 4, *p* < 0.01; *GATA*: t = −6.05, df = 4, *p* < 0.01) (Figure 2P,S). The expression of *4EBP* gene was significantly inhibited (t = 3.16, df = 4, *p* < 0.05) (Figure 2R). However, the expression of *RHEB1*, *RHEB2,* and *S6K2* was not affected (*RHEB1*: t = −1.33, df = 2.09, *p* > 0.05; *RHEB2*: t = −0.70, df = 4, *p* > 0.05; *S6K2*: t = −0.93, df = 4, *p* > 0.05) (Figure 2M,N,Q). The above results showed that 24 h of parasitism affected the signal transduction of IIS pathway, but the effect was not significant and did not affect TOR signal transduction.

### 3.3. Cloning and Expression Analysis of IMP-L2 Gene from S. guani Venom

The aforementioned analyses demonstrated that the parasitism of *S. guani* significantly impacts the IIS/TOR signaling pathway, which is crucial for the growth and development of *T. molitor*. Parasitic wasps are known to impair host growth and development by introducing parasitic factors, including venom. Our previous research identified that the venom of *S. guani* is abundant in the IMP-L2 protein. Consequently, this study focused on cloning and analyzing the *IMP-L2* gene, assessing its expression across various tissues under parasitic and non-parasitic conditions, as well as during different developmental stages. Additionally, we successfully obtained the recombinant protein.

**Figure 3:**
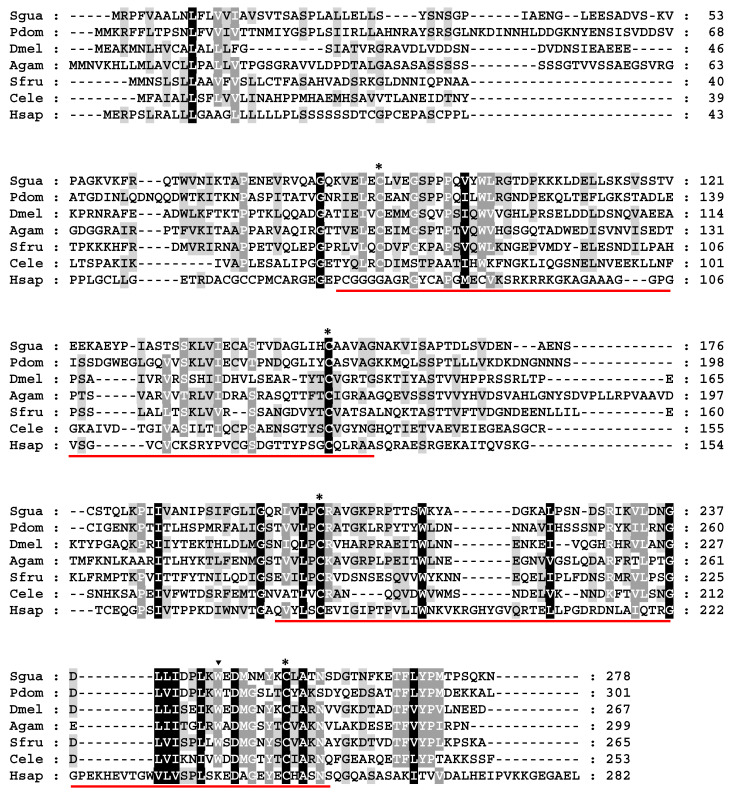
Analysis of multiple alignment of IMP-L2 sequence from *S. guani* venom and other species. Multiple sequence alignment of IMP-L2 in invertebrates and humans. The black shadow represents the identical of residual site, and the gray shadow represents the similar of residual site. The triangle marker IMP-L2 premature termination codon, the asterisk marker is the formation of two disulfide bonds of cysteine, and the red line refers to the IG domain. Sgua: *Sclernderma guani*; Pdom: *Polistes dominula* (XP_015175065.1); Dmel: *Drosophila melanogaster* (Q09024.4); Agam: *Anopheles gambiae* (XP_312831.5); Sfru: *Spodoptera frugiperda* (AAF61949.1); Cele: *Caenorhabditis elegans* (CCD63842.1); Hsap: Homo sapiens, (Q16270.1).

We elucidated the bioinformatics characteristics of *IMP-L2*, particularly its expression in response to *S. guani* parasitism and nutritional status, providing a comprehensive foundation for subsequent functional validations of the IMP-L2 protein. The cloned IMP-L2 gene comprised a coding region of 837 bp, which encodes a protein of 278 amino acids (Figure 3). The identified signal peptide is MRPFVAALNLFLVVIAVSVTSA, with a theoretical molecular weight of 30.01 kDa and an isoelectric point of 5.63 (Figure 3). Notably, an N-glycosylation site (NDS at positions 225–227) was also identified (Figure 3).

Homologous alignment analyses revealed amino acid identities of 38%, 21%, 25%, 25%, 18%, and 13% when compared to the corresponding IMP-L2 proteins from *Polistes dominula*, *Drosophila melanogaster*, *Anopheles gambiae*, *Spodoptera frugiperda*, *Caenorhabditis elegans*, and humans, respectively (Figure 3). A phylogenetic tree constructed using the IMP-L2 proteins from various Hymenoptera species, with *D. melanogaster* as an outgroup, indicated the presence of a single IMP-L2 gene in Ichneumonoidea, Chalcidoidea, and Bethyloidea, while gene expansion was observed in the Vespoidea, Formicidae, and *Apoidea lineages* (Figure S1).

**Figure 4:**
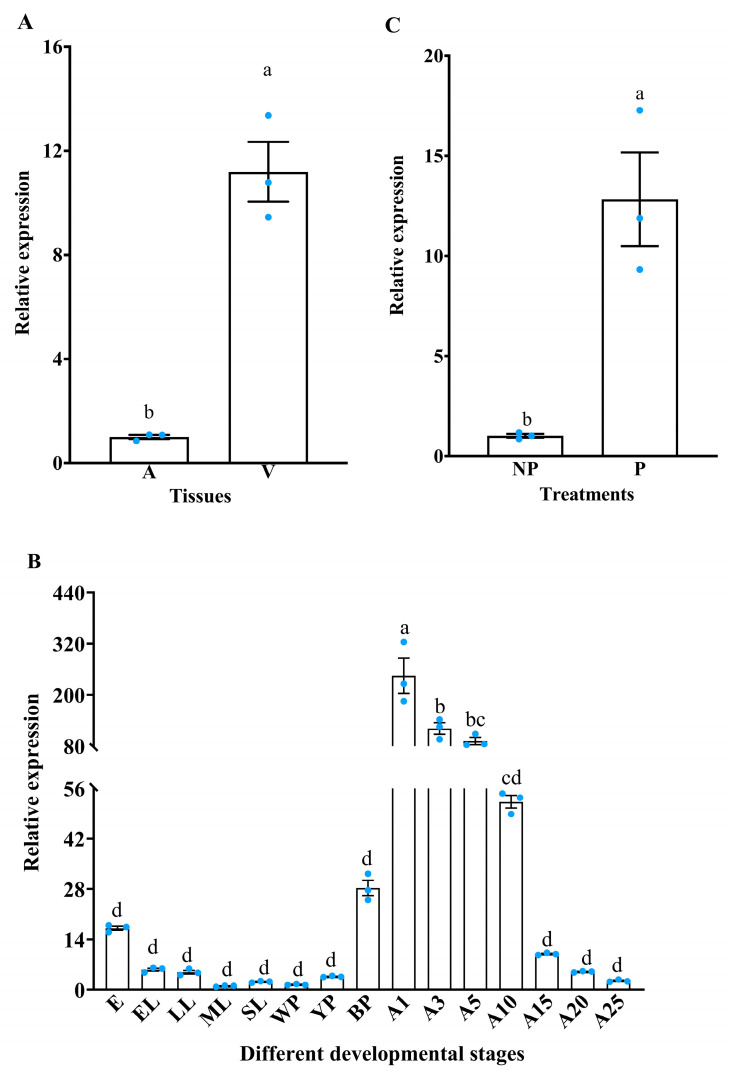
Analysis of venom *IMP-L2* gene expression pattern in *S. guani*. A Expression of *IMP-L2* gene in different tissues. (**A**), abdominal remnants; V, venom apparatus. (**B**) The expression of *IMP-L2* gene at different developmental stages. E, egg; EL, early larvae; LL, late larvae; ML, mature larvae; SL, spinning mature larvae; WP, pupae in white cocoon; YP, pupae in yellow cocoon; BP, pupae in black cocoon; A1, adult after 1 day of emergence; A3, adult after 3 days of emergence; A5, adult after 5 days of emergence; A10, adult after 10 days of emergence; A15, adult after 15 days of emergence; A20, adult after 20 days of emergence; A25, adult after 25 days of emergence. (**C**) Effect of parasitization on expression of *IMP-L2* gene. P, parasitization; NP, non-parasitization. (**A**,**C**) means ± SE within different tissues and different treatments followed by the different lower-case letters are significantly different (*p* < 0.05, Student’s *t* test), while solid circles indicate values for individual biological replicates. (**B**) means ± SE within different tissues and different treatments followed by the different lower-case letter are significantly different (*p* < 0.05, Tukey’s multiple comparison test) while solid circles indicate values for individual biological replicates.

Simultaneously, RT-qPCR analysis revealed that the expression of the *IMP-L2* gene in the venom apparatus of *S. guani* was significantly elevated compared to that in abdominal remnants (t = −8.86, df = 4, *p* < 0.01) (Figure 4A). The *IMP-L2* gene was expressed under different developmental stages (Figure 4B). Notably, the *IMP-L2* gene was highly expressed in adults (especially 1–5 days after eclosion) (F_14,30_ = 37.76, *p* < 0.001), which may be due to the fact that the venom organs of *S.guani* were not formed or not well developed during the egg, larva, and pupa stages, and the development was perfect after eclosion. Therefore, the IMP-L2 gene was highly expressed in adults (Figure 4B). Furthermore, significant upregulation of the *IMP-L2* gene was observed following the parasitism of *T. molitor* pupae by *S. guani* (t = 5.04, df = 4, *p* < 0.01) (Figure 4C), suggesting that the *IMP-L2* gene may play a role in the process of *S. guani* parasitizing *T. molitor* pupae.

**Figure 5:**
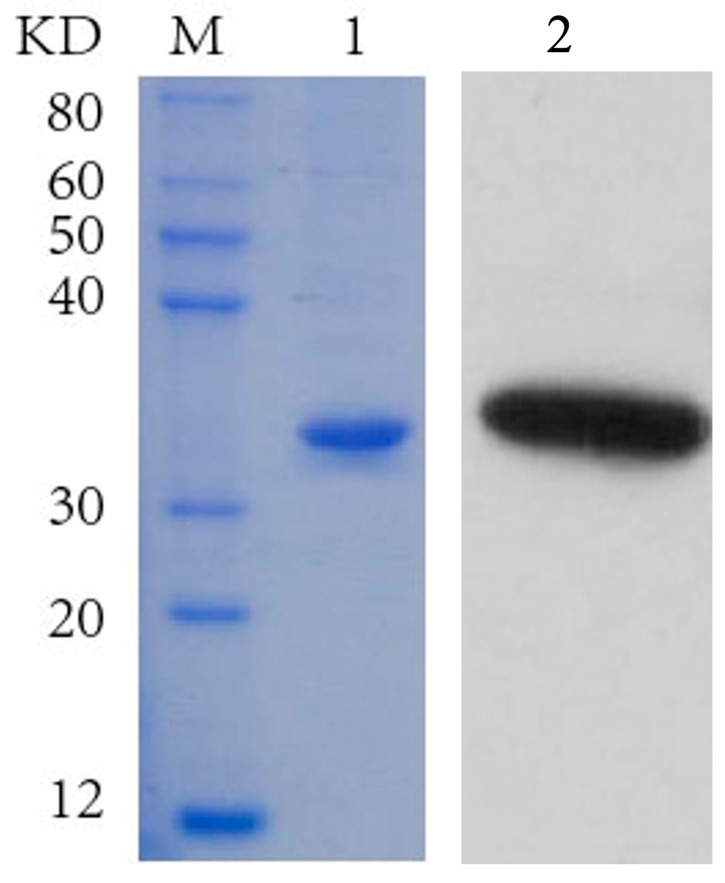
Eukaryotic expression of venom IMP-L2 protein of *S. guani*. M, standard of protein molecular weight; 1, the purified IMP-L2 protein was detected by SDS-PAGE; 2, the purified IMP-L2 protein was detected by Western blot.

The recombinant protein IMP-L2 was successfully produced through eukaryotic expression and subsequent purification. SDS-PAGE analysis revealed that the molecular weight of the recombinant IMP-L2 was approximately 30 kDa, aligning closely with the predicted theoretical molecular weight (Figure 5). Additionally, Western blot results confirmed that the purified protein corresponded to the recombinant IMP-L2 (Figure 5). This recombinant protein is suitable for use in subsequent injection experiments.

### 3.4. Analysis of the Expression Pattern of Genes Related to the IIS/TOR Signaling Pathway in T. molitor After Injection of Recombinant IMP-L2 Protein

The results from the aforementioned experiments indicate that the *IMP-L2* gene is specifically and highly expressed in the venom apparatus and may play a role in parasitism. Previous studies have shown that IMP-L2 from non-venom sources can bind to insulin, inhibiting insulin signal transduction and consequently impeding growth and development [43,45]. To assess whether venom-derived IMP-L2 similarly affects the host, we conducted an injection experiment using recombinant IMP-L2 to evaluate its impact on gene expression within the growth and development-related IIS/TOR pathway.

**Figure 6:**
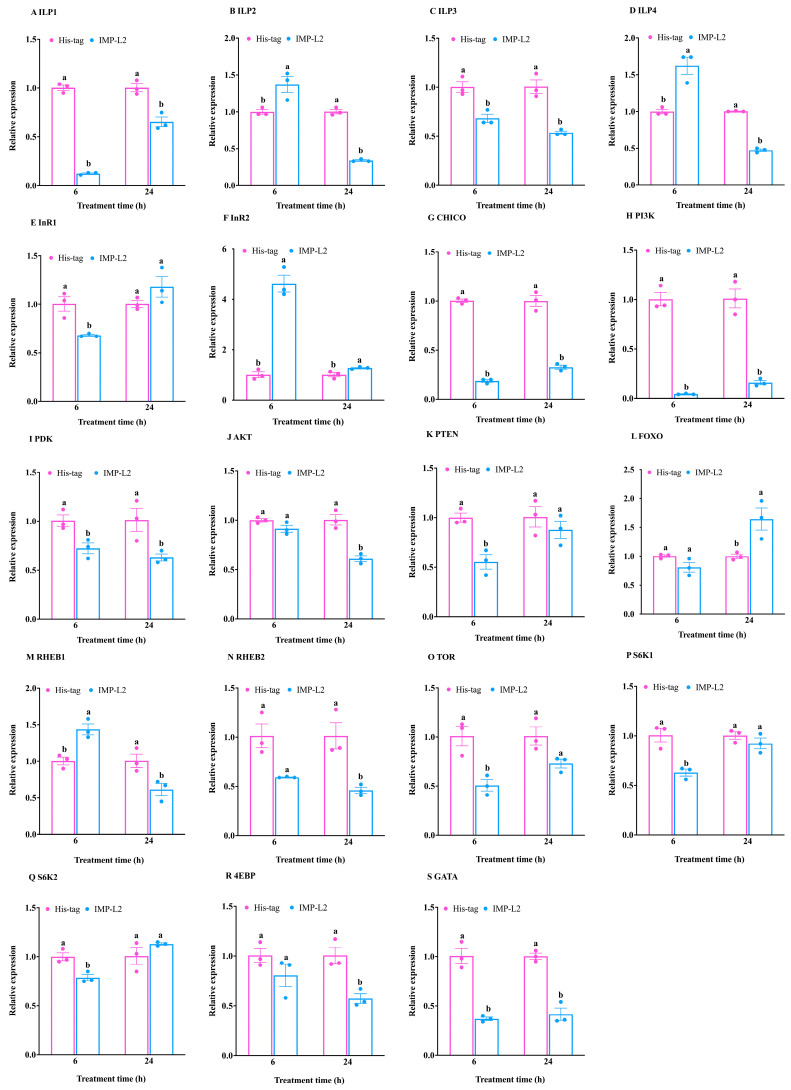
Effect of venom IMP-L2 of *S. guani* on the expression of IIS/TOR related genes of *T. molitor*. (**A**–**D**) Expression of insulin-like peptide (*ILP*)-1/2/3/4; (**E**,**F**) expression of insulin receptor (*InR*)-1/2; (**G**) expression of insulin receptor substrate protein (*CHICO*); (**H**) expression of phosphatidylinositol 3-kinase (*PI3K*); (**I**) expression of phosphoinositide-dependent protein kinase (*PDK*); (**J**) expression of protein kinase B (*AKT*); (**K**) expression of phosphatase and tensin homolog (*PTEN*); (**L**) expression of transcription factor forkhead box class O (*FOXO*); (**M**,**N**) expression of ras homolog enhanced in brain (*RHEB*)-1/2; (**O**) expression of target of rapamycin (*TOR*); (**P**,**Q**) expression of stimulating ribosomal protein S6 kinase (*S6K*)-1/2; (**R**) expression of translational repressor 4e-binding protein (*4EBP*); (**S**) expression of GATA transcription factor (*GATA*) at different time after His-tag injection and IMP-L2 injection. Means ± standard error (means ± SE) corresponding to different letters within a same gene at same time was significantly different (*p* < 0.05, Student’s *t* test) while solid circles indicate values for individual biological replicates.

Six hours post-injection of the IMP-L2 venom protein, the expression levels of the *ILP1* and *ILP3* genes were significantly reduced compared to the control group (*ILP1*: t = −33.43, df = 4, *p* < 0.001; *ILP3*: t = −4.64, df = 4, *p* < 0.05) (Figure 6A,C). Conversely, the expression levels of *ILP2* and *ILP4* genes in the treatment group were significantly increased relative to the control group (*ILP2*: t = 3.35, df = 4, *p* < 0.05; *ILP4*: t = 5.07, df = 2.30, *p* < 0.05) (Figure 6B,D). Additionally, the expression of the *InR2* gene was induced (t = −10.25, df = 4, *p* < 0.01) (Figure 6F), while the expression of host genes *InR1*, *CHICO*, *PI3K*, *PDK,* and *PTEN* was inhibited (*InR1*: t = 4.30, df = 4, *p* < 0.05; *CHICO*: t = 36.94, df = 4, *p* < 0.001; *PI3K*: t = 14.02, df = 2.01, *p* < 0.01; *PDK*: t = 3.54, df = 4, *p* < 0.05; *PTEN*: t = 5.22, df = 4, *p* < 0.01) (Figure 6E,G–I,K).

These findings demonstrate that IMP-L2 inhibits the signal transduction of the InR1/PDK pathway; however, downstream *AKT* expression remained unaffected (t = 2.21, df = 4, *p* > 0.05), as did the transcription of *FOXO* (t = 2.23, df = 4, *p* > 0.05) (Figure 6J,L). Furthermore, the expression of the *RHEB1* gene in the TOR signaling pathway was significantly elevated compared to the control group, while *RHEB2* expression was unchanged (*RHEB1*: t = −4.72, df = 4, *p* < 0.01; *RHEB2*: t = 3.47, df = 2.00, *p* > 0.05) (Figure 6M,N).

In addition, downregulation of *TOR* expression inhibited the transcription of *S6K1*, *S6K2*, and *GATA* genes without affecting *4EBP* expression (*TOR*: t = 4.34, df = 4, *p* < 0.05; *S6K1*: t = 4.90, df = 4, *p* < 0.01; *S6K2*: t = 4.15, df = 4, *p* < 0.05; *GATA*: t = 8.14, df = 4, *p* < 0.01; *4EBP*: t = 1.51, df = 4, *p* > 0.05) (Figure 6O–S). Collectively, these results indicate that the injection of IMP-L2 has a minimal impact on the IIS signaling pathway while significantly inhibiting TOR pathway signal transduction.

The expression of *ILP1*-*ILP4* gene was significantly lower than that in the control group at 24 h after injection of IMP-L2 venom protein (*ILP1*: t = −5.58, df = 4, *p* < 0.01; *ILP2*: t = −22.34, df = 4, *p* < 0.001; *ILP3*: t = −6.52, df = 4, *p* < 0.01; *ILP4*: t = −32.18, df = 4, *p* < 0.001) (Figure 6A–D). The expression levels of *InR2* and *FOXO* genes were significantly increased (*InR2*: t = −3.12, df = 4, *p* < 0.05; *FOXO*: t = −3.30, df = 4, *p* < 0.05) (Figure 6F,L), while the expression of *CHICO*, *PI3K*, *PDK,* and *AKT* was significantly inhibited (*CHICO*: t = 11.47, df = 4, *p* < 0.001; *PI3K*: t = 8.71, df = 4, *p* < 0.01; *PDK*: t = 3.09, df = 4, *p* < 0.05; *AKT*: t = 6.58, df = 4, *p* < 0.01) (Figure 6G–J). The results showed that the venom IMP-L2 affected the IIS signal transduction and activated the FOXO signal by inhibiting the host CHICO/AKT signaling cascade, but the expression of *InR1* and *PTEN* was not affected (*InR1*: t = −1.58, df = 4, *p* > 0.05; *PTEN*: t = 0.97, df = 4, *p* > 0.05) (Figure 6E,K). At the same time, the expression of *RHEB1* and *RHEB2* genes was inhibited (*RHEB1*: t = 3.19, df = 4, *p* < 0.05; *RHEB2*: t = 4.03, df = 2.23, *p* < 0.05) (Figure 6M,N), and then inhibited the expression of *GATA* gene downstream of TOR signaling pathway (t = 8.45, df = 4, *p* < 0.01) (Figure 6S), and inhibited the expression of *4EBP* (t = 4.55, df = 4, *p* < 0.05) (Figure 6R), but did not affect the expression of *TOR*, *S6K1* and *S6K2* genes (*TOR*: t = 2.71, df = 4, *p* > 0.05; *S6K1*: t = 1.21, df = 4, *p* > 0.05; *S6K2*: t = −1.45 df = 4, *p* > 0.05) (Figure 6O–Q). These results suggest that injection of IMP-L2 after 24 h inhibits IIS/TOR pathway signal transduction.

### 3.5. S. guani IMP-L2 Affects the Longevity of T. molitor

**Figure 7:**
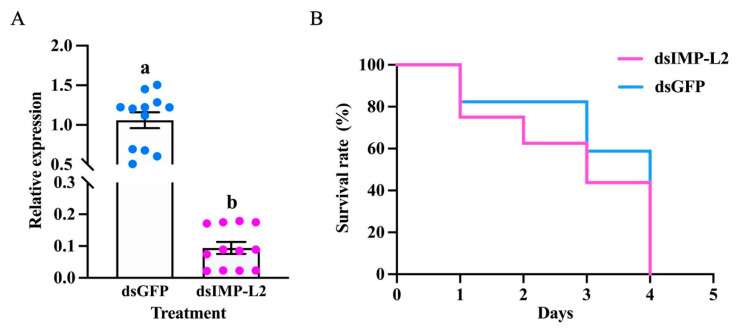
The interference efficiency of *S.guani IMP-L2* (after 24 h) and its effect on the survival of *T. molitor* pupae after silencing. (**A**), the interference efficiency of *IMP-L2* after 24 h of RNAi. (**B**), surviva curve of pupae of *T. molitor* after RNAi. Different lowercase letters indicate significant differences (*p* < 0.05, Student’s *t* test), while solid circles indicate values for individual replicates.

At 24 h after RNAi, compared with dsGFP injection, the relative expression of the *IMP-L2* gene decreased significantly. The expression level of *IMP-L2* gene decreased by 85.47% (Figure 7A). When *S. guani IMP-L2* was silenced and parasitized *T. molitor* pupae, the death rate of *T. molitor* pupae was faster than that of *T. molitor* pupae parasitized by *S. guani* treated with dsGFP (Figure 7B).

## 4. Discussion

The IIS/TOR signaling pathway regulates the developmental stages of *T. molitor*. Our findings indicate that *CHICO*, *PTEN*, *RHEB2*, and *GATA* are primarily involved in embryonic development. Previous studies have linked *CHICO* to embryonic development in *Drosophila*, where *CHICO* mutants extend egg diapause [56]. Similarly, *PTEN* is implicated in the embryonic development of *Aedes aegypti* and *Bombyx mori*, exhibiting high expression during the egg stage [57]. In contrast to our results showing *RHEB2*’s role in *T. molitor* embryogenesis, it appears that *RHEB2* does not influence yolk formation or embryonic development in *Tribolium castaneum* [58]. Furthermore, *Pannier* (*pnr*), a *GATA* family member in *Bactrocera dorsalis*, is essential for both embryonic and post-embryonic development, with knockout leading to significant mortality during these stages [59].

High expression levels of *ILP4*, *PDK*, *RHEB1*, *TOR*, and *S6K2* during larval development suggest their functional roles at this stage. *ILP4* similarly influences larval development in *Gnatocerus cornutus*, where *GcorILP4* is highly expressed in the larval stage [60]. *PDK* is predominantly expressed during late larval stages, potentially aiding prepupal development [61]. Notably, *RHEB1*’s involvement extends beyond yolk development to include oviposition, cell volume regulation, neuronal function, and lifespan [58,62,63]. Our findings align with previous research indicating that *TOR* is crucial for *Drosophila* larval development, with reduced activity resulting in larval mortality [14]. Moreover, *S6K2*’s role in larval growth and development highlights its additional functions beyond egg development [64,65].

We observed that IIS pathway-related genes are critically important during the prepupal and pupal stages, with seven genes (*ILP1*, *ILP2*, *ILP3*, *InR1*, *PI3K*, *PDK*, *AKT*) identified. *GCORILP3* in *G. cornutus* and *Mv-ILP1* in *Maruca vitrata* also exhibit high expression during these stages [60,66]. Interestingly, *ILP2* showed peak expression during the prepupal stage, contrasting with its predominance in insect larvae and adults [67]. Consistent with our findings, *InR*, *PI3K*, *PDK,* and *AKT* are also highly expressed during the prepupal stage in *Bombyx* [61]. These results suggest that the prepupal stage of *T. molitor* primarily relies on the IIS pathway for growth and development.

*InR* and *FOXO* affect ovarian development and oviposition in adults, and *TOR*, *S6K1,* and *4EBP* are involved in vitellogenesis in adults [68,69,70]. The significant upregulation of these genes in *T.molitor* may be involved in the regulation of these physiological processes.

At early stage (6 h post-parasitism), the expressions of *ILP1* and *ILP3* were suppressed, while *ILP4* expression appeared compensatory, leading to an increase in *ILP1* and *ILP3* levels, consistent with observations in *Drosophila* [71]. The upregulation of *InR2* did not inhibit *InR1* expression, resulting in unaffected downstream *CHICO* expression. Although PI3K/PDK signaling was inhibited, *AKT* expression was ultimately upregulated, indicating that IIS signals were still transmitted to the TOR pathway. This upregulation may be attributed to the activation of the ROS/AKT/S6K/CREB/HIF1 pathway, which regulates pupal diapause during parasitic stages, thereby compensating for the inhibitory effects on *AKT* expression stemming from PI3K/PDK/AKT signaling inhibition [72].

Concurrently, *RHEB1* of the TOR pathway was upregulated in response to increased *AKT* levels. However, *TOR* complex expression was suppressed at 6 h post-parasitism, which inhibited TOR signaling and consequently delayed the growth and development of *T. molitor*. This inhibition of the TOR pathway may induce a state akin to “starvation restriction,” which is known to prolong lifespan and reduce fecundity in various organisms, similar to effects observed in *Drosophila* [73,74].

At 24 h after parasitism, *ILP1*, *ILP3,* and *ILP4* expressions remained suppressed, while *ILP2* was upregulated, potentially in response to IMP-L2 induction, analogous to the upregulation of *DILP2* in *Drosophila* following IMP-L2 overexpression [43]. Despite significant *InR2* expression inhibiting InR1/CHICO signaling, *PTEN* was also upregulated, yet it did not impact PI3K/PDK/AKT signaling, leaving TOR signaling unaffected. This suggests that the inhibitory effects of parasitic factors on the IIS/TOR pathway may diminish following host consumption and metabolism, leading to a gradual restoration of IIS/TOR signaling.

The insulin-binding protein IMP-L2 is crucial in Hymenoptera species, exhibiting high expression levels specifically within the venom apparatus of *S. guani*. The cloned IMP-L2 from *S. guani* possesses an immunoglobulin domain that shows significant homology with IMP-L2 from other organisms, suggesting a common ancestral origin as an insulin-binding protein [38,39]. We found that gene duplication may occur in the common ancestor of the post-differentiated species, and the phenomenon of gene number amplification occurs in the post-differentiated species.

Additionally, the elevated expression of *IMP-L2* in the venom apparatus corroborates our earlier findings that identified this protein as prevalent in the venom of *S. guani* [50]. Both IMP-L2 and its homolog EpIBP have been detected in the venoms of *N. vitripennis* and *E. pomiformis*. In *E. pomiformis*, EpIBP serves as a primary venom component that interacts with the apolipophorin III (apoLp III) protein of Lepidoptera, influencing host metabolism through this interaction [51,52]. It is plausible that IMP-L2 in *S. guani* may exhibit analogous functions.

The venom-derived IMP-L2 from *S. guani* is induced during parasitism, potentially facilitating the delay of host development and lifespan extension by inhibiting IIS and the TOR pathway during the parasitism of *T. molitor*. The *IMP-L2* gene is highly expressed in parasitized *S. guani*, likely due to transcription activation coinciding with venom secretion via the sting, suggesting its functional role in the parasitic process.

Following the injection of recombinant IMP-L2, inhibition of host IIS/TOR signal transduction was observed at both 6 h and 24 h post-injection. Consistent with the parasitism results, the expression of *ILP1* and *ILP3* was suppressed at 6 h, while *ILP2* showed compensatory expression alongside *ILP4*. Notably, increased *InR2* expression inhibited the entire InR1/Chico/PI3K/PDK pathway, although *AKT* levels remained comparable to controls. This suggests that compensatory AKT activation within the pupal diapause-related pathway offsets the reduction in AKT associated with the IIS signaling pathway.

In contrast to parasitism, IMP-L2’s inhibition of the TOR complex resulted in a broader impact on downstream gene expression. The venom composition of parasitic wasps is complex, and the concentration of free amino acids in the hemolymph of the host will increase after the venom is injected into the host [75,76]. High concentrations of amino acids can stimulate the TOR signaling cascade [30]. Therefore, the reason why the inhibitory effect of TOR signaling pathway caused by parasitism is not as good as that of pure IMP-L2 may be that high concentration of amino acids stimulation TOR signaling cascade offsets the inhibitory effect of some IMP-L2. At 24 h post-injection, *ILP1-4* expression was down-regulated, mirroring results observed during parasitism for *ILP1*, *ILP3,* and *ILP4*. The upregulation of *InR2* was linked to the suppression of signaling throughout the InR1/Chico/PI3K/PDK/AKT pathway, while significant downregulation of *AKT* activated *FOXO* transcription, potentially triggering diapause in pupae.

The lack of compensation for the downregulation of *ILP2* and *AKT* at 24 h indicates a robust inhibition of IIS signaling. In *Drosophila*, IIS pathway suppression results in reduced food intake, extended lifespan, and decreased fecundity, suggesting similar effects may occur in *T. molitor* due to IIS pathway inhibition. Additionally, at 24 h post-injection, IMP-L2 inhibited the TOR pathway in *T. molitor*, primarily affecting the expression of *RHEB1/2* and *GATA*. Collectively, these results imply that *S. guani* predominantly targets the host IIS pathway through venom-derived IMP-L2 at the 24 h mark, with high concentrations of IMP-L2 exerting potent inhibitory effects on both the IIS and TOR signaling pathways.

In addition, we found that after *IMP-L2* knockdown, the pupae of *T. molitor* parasitized by *S. guani* accelerated death and the death time was shortened. This result indicates that IMP-L2 affects the lifespan of *T. molitor*. It may be that *IMP-L2* knockdown reduces the binding of ILP, restores the signal transduction of IIS/TOR pathway, accelerates nutrient consumption, and accelerates its death.

## 5. Conclusions

In conclusion, the IIS/TOR pathway is crucial for regulating the growth and development of all stages of *T. molitor*. In the initial phase (6 h) following parasitism, *S. guani* modulates the growth, development, and longevity of *T. molitor* by targeting the TOR signaling pathway. By the later stage (24 h) post-parasitism, host IIS/TOR signal transduction begins to recover as parasitic factors diminish. Notably, the venom of *S. guani* is particularly enriched in the insulin-binding protein IMP-L2, which is vital for Hymenoptera insects, and responds to parasitic stimuli.

In the early phase post-injection, the strategy mirrors that of early parasitism, wherein IMP-L2 inhibits the host TOR signaling pathway, albeit with a more pronounced effect at higher concentrations. This inhibition extends into the late stage of injection, demonstrating that *S. guani* primarily employs venom-derived IMP-L2 to impede the IIS/TOR signaling pathway, thereby delaying host growth and development. In addition, the absence of IMP-L2 accelerates the death of the host. This mechanism ultimately prolongs the host’s lifespan, facilitating the successful development of parasitic wasp eggs and larvae.

## Data Availability

The data presented in this study are openly available in NCBI at (https://www.ncbi.nlm.nih.gov/genbank/, accessed on 5 October 2024). The accession numbers for each dataset are provided in Table S1.

## Supplementary Materials

The following supporting information can be downloaded at: https://www.mdpi.com/article/10.3390/insects16080763/s1, Figure S1: Phylogenetic tree of IMP-L2 sequences from venom of *Scleroderma guani* and other species; Figure S2: Original Western blot images for Figure 5; Table S1: The primer for RT-PCR or RT-qPCR of related genes of IMP-L2 and IIS/TOR pathway from *Scleroderma guani* or *Tenebrio molitor* respectally.
