# Supplementary material for: Venom IMP-L2 from the Ectoparasitoid Scleroderma guani Regulates the IIS/TOR Pathway in Tenebrio molitor
Source: Insects. 2025 Jul 24;16(8):763. doi: 10.3390/insects16080763 (PMC12386756; doi:10.3390/insects16080763)
Supplement: Supplementary file 1 [file insects-16-00763-s001.zip › insects-3736500-supplementary.pdf]

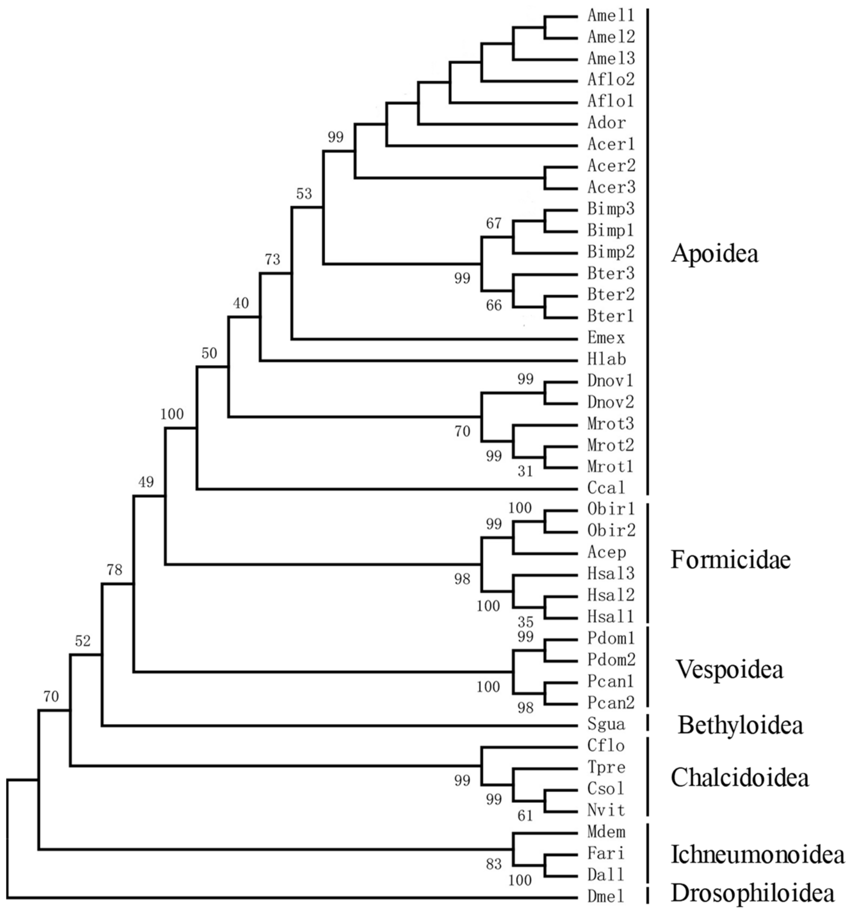

Figure S1 Phylogenetic tree of IMP-L2 sequences from venom of *Scleroderma guani* and other species. Apidae: Amel1-3: *Apis mellifera* (XP_016770571.1) (XP_006564095.1) (XP_006564096.1); Ador: *Apis dorsata* (XP_006608037.1); Acer1-3: *Apis cerana* (XP_016919478.1) (XP_016919479.1) (XP_016919480.1); Aflo1-2: *Apis florea* (XP_012350714.1) (XP_012350715.1); Bimp1-3: *Bombus impatiens* (XP_003489640.1) (XP_012241183.1) (XP_024223495.1); Bter1-3:

Bombus terrestris (XP_012168786.1) (XP_003398836.1) (XP_020721065.1); Emex: *Eufriesea mexicana* (OAD61856.1); Ccal: *Ceratina calcarata* (XP_017883957.1); Hlab: *Habropoda laboriosa* (KOC69633.1): Halictidae: Dnov1-2: *Dufourea novaeangliae* (XP_015436601.1) (KZC13838.1); Megachilidae: Mrot1-3: *Megachile rotundata* (XP_012135289.1) (XP_003700985.1) (XP_ 012135290.1). Vespoidea: Pcan1-2: *Polistes canadensis* (XP_014611278.1) (XP_014611279.1); Pdom1-2: *Polistes dominula* (XP_015175064.1) (XP_015175065.1). Bethylidea: Sgua: *Sclernderma guani*. Chalcidoidea: Encyrtidae: Cflo: *Copidosoma floridanum* (XP_023245024.1); Agaonidae: Csol: *Ceratosolen solmsi* (XP_011498175.1); Pteromalidae: Nvit: *Nasonia vitripennis*, (XP_001608198.2); Trichogrammatidae: Tpre: *Trichogramma pretiosum* (XP_014236860.1). Ichneumonoidea: Braconidae: Dall: *Diachasma alloeum* (XP_015122574.1); Fari: *Fopius arisanus* (XP_011313218.1); Mdem: *Microplitis demolitor* (XP_008559902.1). Formicidae: Dorylinae: Obir1-2: *Ooceraea biroi* (XP_011333209.1) (EZA57702.1); Ponerinae: Hsal1-3: *Harpegnathos saltator* (EFN84233.1) (XP_011139853.1) (XP_011139855.1); Myrmicinae: Acep: *Atta cephalotes* (XP_012054672.1). Drosophiloidae: Drosophilidae: Dmel: *Drosophila melanogaster* (Q09024.4).

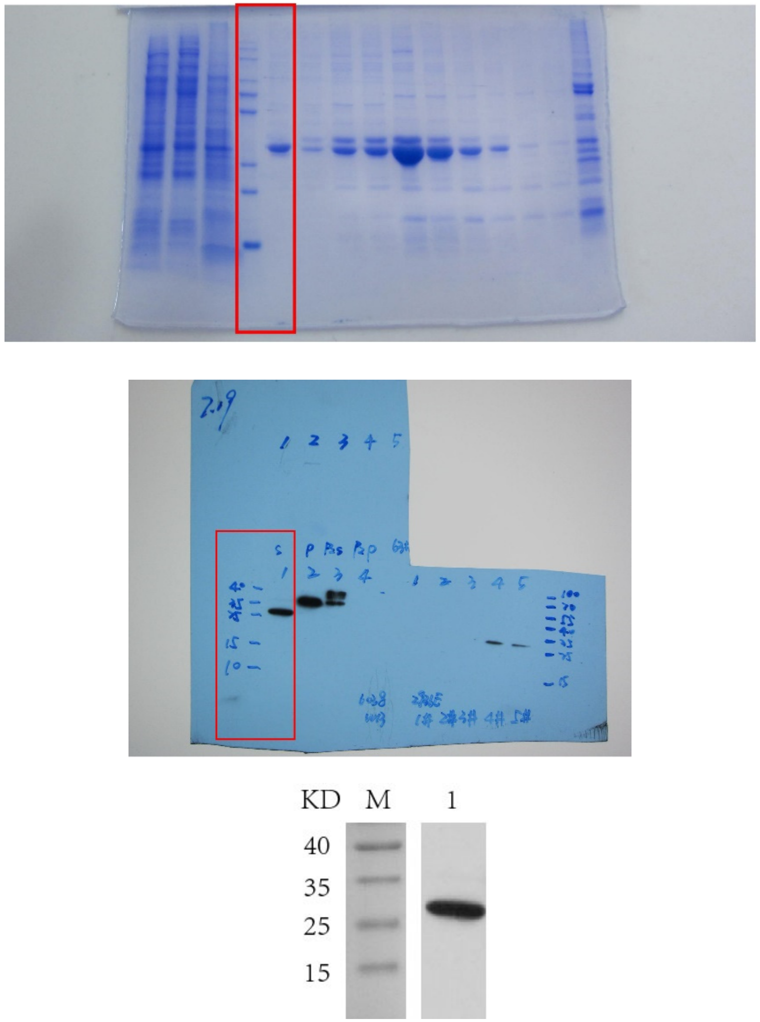

Figure S2 Original Western blot images for Figure 5.

Table S1 The primer for RT-PCR or RT-qPCR of related genes of IMP-L2 and IIS / TOR pathway from *Scleroderma guani* or *Tenebrio molitor* respectally

Gene name (Genbank)	5’-3’Forward primer	5’-3’ Reverse primer
Construction of expression vector		
pFast-bac1-*IMP-L2*	TTCATACCGTCCCACCATCGGCGCGGATCCGCCACCA TGCTGCTGGTGAACCAGTCC	TTATGATCCTCTAGTACTTCTCGACAAGCTTTTAATGGT GGTGGTGGTGGTGATGGTTCTT
RT-PCR		
IMP-L2 (PP108620)	ATGAGGCCTTTCGTAGCTGCATTGAAT	GCTTGGAGTCATGGGGTAAAGGAAAGT
RT-qPCR		
Rpl32 (PQ231177)	GGACCGTTATGGCAAACTCA	GAGCATGTGTCTCGTTTTGC
ILP1 (PP108621)	TGTATGGTGTACACCTCATATGGC	CATTCTCACTGGGGAAAAGCTCGT
ILP2 (PP108622)	GGCTCGCAGAACAATAAGAAGA	GTTGTAGTTTCCCTTGCAGACG
ILP3 (PP108623)	CAACAATCCGACCTATGC	CCGAACTCTTTGCTTCAG
ILP4 (PP108624)	GATCACGTCGGAATGTTG	TGTATCTCTTGTTGGTTGGA
InR1 (PP108625)	CACGCAACCAACAGGGAA	CTGGACGGTGAGAACGCA
InR2 (PP108626)	GGAATAATAACCACTGCCAAGA	TCCACCAACTACCGCTGA
CHICO (PQ231178)	CATCACCAGCATCCAAGA	GTAGAGAACAGTCCCATCGT
PDK (PP108628)	CACATCATCAGGGAGAAA	AAGCAGGTGTCCTTATTG
AKT (PP108629)	GAAGGAGGGATGGCTGTT	TGAAAGTGAAGGGTTTAGGT
PI3K (PP108627)	AGTGCCTTGGAAATGAAC	TGGACAAAGTCTTGGTGG
PTEN (PP108631)	CACCTCTTTCCCCAACATGA	TGCTTCGAGTCGAGGAGCTT
FOXO (PP108630)	GAGACTGACGTTGTCGCAGA	CGGGATTAATCATCCACCAG
RHEB1 (PP108632)	CCTTAGCATTCAGTTTGTT	CGTGAGTTCATAATCTTGTG
RHEB2 (PP108633)	GGCAAGTTCCTCGAGTCGTA	GTAGTTTTTGCCGGTGATGT
TOR (PP108634)	CTAATGAGAAGAAGGGTGG	ATACTCTGAGGCTTTGGAC
S6K1 PQ231179	GCGGACACGGCAAAGAGGT	TCAGATACGGCGGCAGGA

Gene name (Genbank)	5'-3'Forward primer	5'-3' Reverse primer
S6K2 (PP108636)	TGCCGTTCCAAGGTAGTA	AGTCGGTTAGCAGGATTTC
4EBP (PP108635)	GAGTTACGCAAGTTCATCAGG	GCCAAGTCACCGAAAGCA
GATA (PP108637)	ATTCTAACGCAGGGTTGGTG	ACTAGCGGGCTCGGTGTATT
IMP-L2 (PP108620)	GGCAAAGTGAAATTCCGACAG	CGCTCTTACTCAGAAGCTCGTC
5.8 S rRNA (FJ603076.1)	AAGAGCGACGCCAAACC	ATGGGTCACTCGACTGGAT
18 S rRNA (PQ231176)	TGGGCCGGTACGTTTACTTT	CACCTCTAACGTCGCAATAC
Synthesis of dsRNA		
dsIMP-L2-F/ T7-dsIMP-R	ATGAGGCCTTTCGTAGCTGCATTG	TAATACGACTCACTATAGGCTGCAGCTGTTTTCGGCATTTTCATC
T7-dsIMP-F/ dsIMP-R	TAATACGACTCACTATAGGATGAGGCCTTTCGTAGCTGCATTG	CTGCAGCTGTTTTCGGCATTTTCATC
RNAi (RT-qPCR)		
IMP-L2 (PP108620)	TGAAGCCAATCATCGTAG	TTTGATCCGCGAGTCGTTG
5.8 S rRNA (FJ603076.1)	AAGAGCGACGCCAAACC	ATGGGTCACTCGACTGGAT

The underline base is the digestion site (F: BamH I; R: Hind III).
